# Periodontitis and health‐related quality of life in hemodialysis patients

**DOI:** 10.1002/cre2.50

**Published:** 2016-11-24

**Authors:** Masanori Iwasaki, Wenche S. Borgnakke, Shuji Awano, Akihiro Yoshida, Tomoko Hamasaki, Gou Teratani, Shota Kataoka, Satoko Kakuta, Inho Soh, Toshihiro Ansai, Hidetoshi Nakamura

**Affiliations:** ^1^ Division of Community Oral Health Development Kyushu Dental University Kitakyushu Japan; ^2^ Department of Periodontics and Oral Medicine University of Michigan School of Dentistry Ann Arbor Michigan USA; ^3^ Department of Comprehensive Education Kyushu Dental University Kitakyushu Japan; ^4^ Department of Oral Microbiology Matsumoto Dental University Shiojiri Japan; ^5^ Department of Nutrition Faculty of Home Economics Kyushu Women's University Kitakyushu Japan; ^6^ Mikan Dental Clinic Matsuyama Japan; ^7^ Kokura Daiichi Hospital Kitakyushu Japan

**Keywords:** dialysis, cross‐sectional studies, oral health, kidney diseases

## Abstract

Periodontitis is common among dialysis patients. The current cross‐sectional study aimed to explore associations between periodontitis and health‐related quality of life (HRQoL) among hemodialysis patients**.** Data from 188 dentate patients undergoing hemodialysis between May and July 2008 at a medical center in Kitakyushu city, Japan, were analyzed while applying modified Centers for Disease Control and Prevention/American Academy of Periodontology periodontitis case definitions to categorize the participants into the following three groups: severe, moderate, and no/mild periodontitis, respectively. HRQoL was assessed by the Medical Outcomes Study 36‐Item Short Form Health Survey (SF‐36) where a higher score indicates better health status. Associations between periodontitis groups and the eight health domains of SF‐36 were evaluated using general linear models that were adjusted for age, sex, underlying cause of dialysis, duration of dialysis, comorbidities, serum biomarkers, body mass index, smoking status, and alcohol use. Among the 188 participants, 18 (9.6%) had severe periodontitis, 100 (53.2%) had moderate periodontitis, and the remaining 70 (37.2%) had no/mild periodontitis. Compared with the participants with no/mild periodontitis, those with severe periodontitis had worse scores in the following five of eight SF‐36 health scales: physical functioning, role physical, vitality, social functioning, and mental health (*P* < 0.05). The findings suggest an independent relationship between severe periodontitis and decreased HRQoL among dialysis patients.

## 1 INTRODUCTION

The estimated number of dialysis patients in Japan was over 300,000 in 2010, and this number has continued to increase, reflecting increasing prevalence of diabetes and hypertension (Wakasugi, Kazama, & Narita, 2015). Accumulating evidence suggests that decreased kidney function may be associated with poor oral health, including periodontitis (Sharma et al., 2014; Teratani et al., 2013). Sharma et al. compared oral health status in people with chronic kidney disease participating in an ongoing observational cohort study (the Renal Impairment in Secondary Care study) and a regional representative population‐based sample of participants in the 2009 Adult Dental Health Survey. They found that Renal Impairment in Secondary Care participants had a significantly higher risk of periodontitis compared with Adult Dental Health Survey participants (Sharma et al., 2014). Similarly, Teratani et al*.* found that hemodialysis patients had poorer periodontal health than a regional representative population‐based sample (Teratani et al., 2013).

Oral diseases frequently constitute a chronic state and are directly and indirectly associated with physical, psychological, and social functioning; therefore, they can have negative impacts on patients' quality of life (Kandelman, Petersen, & Ueda, 2008; Hollister & Weintraub, 1993; Chen & Hunter, 1996; Buset et al., 2016). Previous studies reported that oral diseases were significantly associated with health‐related quality of life (HRQoL) among patients attending private dental offices (Naito et al., 2010), community‐based older individuals (Wang, Chou, & Yu, 2013; Ng & Leung, 2006), or individuals with diseases related to oral health, such as diabetes (Sandberg & Wikblad, 2003) and chronic obstructive pulmonary disease (Zhou, Wang, Song, Zhang, & Wang, 2011). However, the impact of periodontitis on general HRQoL among dialysis patients has not yet been fully investigated. HRQoL is an important marker of how disease affects patients' lives. Because worse general HRQoL in dialysis patients was found to be associated with greater morbidity and mortality (Lowrie, Curtin, LePain, & Schatell, 2003), it is important to investigate the factors associated with general HRQoL. If an association between periodontal health and general HRQoL was found, such information in turn may lead to new strategies to maintain or improve patients' wellbeing through appropriate oral health education, prevention, and treatment programs. Therefore, this study was planned with the purpose of assessing whether periodontitis was associated with general HRQoL among hemodialysis patients.

## 2 METHODS

### 2.1 Study population and examination

This study was designed as a cross‐sectional survey and conducted at a single medical institution in Kitakyushu city, Japan. Between May and July 2008, 347 sequential patients undergoing hemodialysis were invited to participate in the study. Of these, 221 (63.7% [221/347]) agreed. These individuals underwent oral examination just before a session of hemodialysis therapy and were surveyed using a questionnaire about HRQoL and health behavior. All study participants provided written informed consent to participate in the study.

This study was conducted in accordance with the guidelines laid down in the Declaration of Helsinki and was approved by the Ethics Committee of Kyushu Dental University.

Periodontal examinations were performed by four calibrated dentists. The periodontal health parameters including clinical attachment loss (CAL) and periodontal probing depth (PPD), were measured by placing a periodontal probe at the mesio‐buccal and mid‐buccal sites of each tooth, except for third molars and root remnants. The examiners were calibrated before the survey using volunteer patients in the Kyushu Dental University Hospital. The interexaminer reliabilities of CAL and PPD within 1 mm were assessed using percentage agreement (CAL = 83.3% to 87.5%, PPD = 86.6% to 95.9%).

Using modified Centers for Disease Control and Prevention/American Academy of Periodontology case definitions (Page & Eke, 2007), study participants were categorized into three groups: severe periodontitis was defined as ≥2 sites with CAL ≥ 6 mm (not on the same tooth) and ≥1 site with PD ≥ 5 mm; moderate periodontitis was defined as ≥2 sites with CAL ≥ 4 mm (not on the same tooth), or ≥2 sites with PD ≥ 5 mm (not on same tooth); and no/mild periodontitis was defined as no evidence of severe or moderate periodontitis. The modification was necessary because the Centers for Disease Control and Prevention/American Academy of Periodontology periodontitis case definitions use exactly the four proximal sites, but we only examined one proximal (mesio‐buccal) and one mid‐buccal site.

HRQoL was assessed using the Medical Outcomes Study 36‐Item Short Form Health Survey (SF‐36) version 2, which is a commonly used generic HRQoL scale. The validity of the Japanese SF‐36 is reported elsewhere (Fukuhara, Ware, Kosinski, Wada, & Gandek, 1998). It is designed to assess the following eight health scales: physical functioning (PF), role physical (RP), bodily pain (BP), general health (GH), vitality (VT), social functioning (SF), role emotional (RE), and mental health (MH). Each scale yields a score of 0 to 100, where a high score indicates better health/ a more favorable health state. Detailed descriptions of each SF‐36 scale are presented in Appendix Table 1. The raw scores of eight health scales can be converted into Norm‐Based Scores (NBSs) using Japanese norm‐based scoring algorithms. NBS is a normalized score with a mean of 50 and standard deviation of 10 (Fukuhara & Suzukamo, 2004). The use of the NBS simplifies the interpretation of the results of each subscale of the SF‐36 because it eliminates the need to evaluate the difference between the raw score and national standard for each subscale.

A standardized questionnaire was used to collect data regarding participants' health behavior including smoking status and alcohol consumption. Information on participants' age, sex, underlying cause of hemodialysis, duration of hemodialysis, comorbidities (hypertension, diabetes, depression, ischemic heart disease, and stroke), levels of serum biomarkers (albumin and non‐high‐density lipoprotein cholesterol [non‐HDL‐C]), and body mass index (BMI) were abstracted from medical records. Hypoalbuminemia was defined as albumin <3.6 g/dL. High non‐HDL‐C was defined as non‐HDL‐C ≥ 150 mg/dL.

### 2.2 Statistical analyses

Comparisons of selected characteristics among the three clinically assessed periodontitis groups were performed using analysis of variance or Kruskal–Wallis test for continuous variables, depending on distribution, and the Chi‐square test for categorical variables.

Univariable and multivariable analyses of the association between periodontitis and eight health scales of SF‐36 were conducted using general linear models. The eight health scales of SF‐36, which were converted into NBSs, were included as the main outcomes for the analyses. The principal exposure variable was periodontal health status based on a clinical periodontal examination. Comprehensive multivariable models for the eight health scales included adjustment for age (continuous), sex (categories: male or female), underlying cause of dialysis (categories: diabetic nephropathy, chronic glomerulonephritis, or other), duration of dialysis (continuous), comorbidities (categories: positive or negative), abnormality in serum biomarkers (categories: positive or negative), BMI (continuous), smoking status (categories: current smoker, previous smoker, or never smoked), and alcohol use (categories: ≥20 g/day or <20 g/day ethanol). Effect modifications by sex were evaluated using interaction terms. Interaction terms found not to be statistically significant were not included in the model.

The level of significance for predictor variables was set at α = 0.05. All calculations and statistical analyses were performed using the statistical software package STATA (version 14) (Stata Corp., TX, USA).

## 3 RESULTS

Among those who entered the study (*n* = 221), 24 individuals who had <2 teeth and nine individuals who had incomplete data were excluded. Ultimately, data from 188 hemodialysis patients (age range = 28 to 93 years) were included in the analyses. Among these 188 study participants, 18 (9.6%) were classified as having severe periodontitis, 100 (53.2%) had moderate periodontitis, and the remaining 70 (37.2%) had no/mild periodontitis.

The characteristics of the study population stratified by periodontal health status are presented in Table 1. Besides the lower number of teeth and poorer periodontal health parameters, age, diabetes, and smoking were significantly associated with periodontitis (*P* < 0.05).

**Table 1:** Characteristics of the study participants by periodontitis status (*n* = 188)

	Overall	Periodontitis category			
		No/mild	Moderate	Severe	
	*n* = 188	*n* = 70	*n* = 100	*n* = 18	*P* a
Oral health status					
Number of teeth, median (IQR)	24 (15–27)	26 (21–28)	23 (12–26)	17 (11–25)	**<**0.01*
Mean PPD (mm), median (IQR)	1.7 (1.5–2.0)	1.6 (1.4–1.7)	1.8 (1.6–2.0)	2.4 (2.1–2.8)	**<**0.01*
Mean CAL (mm), median (IQR)	2.4 (1.1–3.5)	1.0 (0.3–1.5)	2.8 (2.2–3.7)	4.1 (3.6–4.9)	**<**0.01*
Age, mean (SD)	63.6 (12.8)	60.5 (13.6)	65.9 (12.3)	63.1 (10.6)	0.03*
Sex, *n* (%)					
Male	118 (62.8)	37 (52.9)	67 (67.0)	14 (77.8)	0.07
Female	70 (37.2)	33 (47.1)	33 (33.0)	4 (22.2)	
Health status and health behavior					
Underlying cause of hemodialysis, *n* (%)					
Diabetic nephropathy	48 (25.5)	12 (17.1)	30 (30.0)	6 (33.3)	0.24
Chronic glomerulonephritis	99 (52.7)	44 (62.9)	47 (47.0)	8 (44.4)	
Others	41 (21.8)	14 (20.0)	23 (23.0)	4 (22.2)	
Duration of hemodialysis (year), median (IQR)	7 (3–16)	8.5 (4–17)	6 (3–16)	5.5 (2–14)	0.49
Medical diagnosis, *n* (%)					
Hypertension	71 (37.7)	30 (42.9)	37 (37.0)	4 (22.2)	0.27
Diabetes	65 (34.6)	15 (21.4)	41 (41.0)	9 (50.0)	0.01*
Depression	5 (2.7)	2 (2.9)	2 (2.0)	1 (5.6)	0.68
Medical history, *n* (%)					
Ischemic heart disease	32 (17.0)	11 (15.7)	18 (18.0)	3 (16.7)	0.93
Stroke	42 (22.3)	14 (20.0)	22 (22.0)	6 (33.3)	0.48
Serum biomarker levels					
Albumin < 3.6 g/dL	73 (38.8)	22 (31.4)	44 (44.0)	7 (38.9)	0.25
Non‐high‐density lipoprotein cholesterol ≥ 150 mg/dL	34 (18.1)	15 (21.4)	18 (18.0)	1 (5.6)	0.30
BMI (kg/m^2^), median (IQR)	20.7 (18.9–22.3)	20.7 (18.8–22.3)	20.7 (19.1–22.5)	19.8 (18.4–21.3)	0.50
Smoking status, *n* (%)					
Current smoker	29 (15.4)	7 (10.0)	18 (18.0)	4 (22.2)	0.04*
Previous smoker	73 (38.8)	21 (30.0)	43 (43.0)	9 (50.0)	
Never smoked	86 (45.7)	42 (60.0)	39 (39.0)	5 (27.8)	
Alcohol consumption, *n* (%)					
Ethanol ≥ 20 g/day	30 (16.0)	10 (14.3)	16 (16.0)	4 (22.2)	0.71

*Note*. BMI = body mass index; CAL = clinical attachment loss; IQR = interquartile range; PPD = periodontal probing depth; SD = standard deviation.

a *P* value for the comparison among the three periodontitis groups.

* *P* < 0.05.

**Figure 1:**
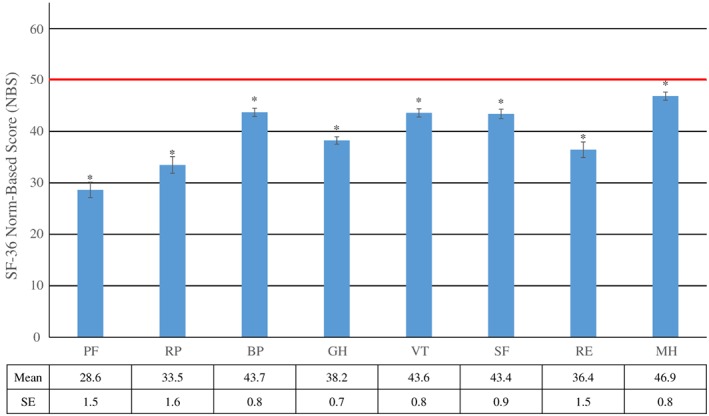
SF‐36 Norm‐Based Score (NBS) by health scale [Mean (SE)] (*n* = 188). The norm among Japanese adults is indicated by red line. BP = bodily pain; GH = general health; MH = mental health; PF = physical functioning; RE = role emotional; RP = role physical; SE = standard error; SF = social functioning; SF‐36 = Medical Outcome Study Short‐Form 36‐item health survey; VT = vitality. Vertical bars represent SE. *Significantly different from the norm of 50 in Japanese adults adults (*p* < 0.05)

SF‐36 health profiles of study participants are presented in Figure 1. Study participants had lower HRQoL than the general Japanese population. Mean SF‐36 scores of study participants in this study were significantly (*P* < 0.05) lower than Japanese norms, which were presented in the Manual of the SF‐36 Japanese version (Fukuhara & Suzukamo, 2004), for all eight scales measured in this study.

Table 2 shows the association between periodontitis and the eight health scales of SF‐36. There were no interactions of periodontitis with sex. Furthermore, when we performed analyses stratified by sex, the pattern of results was similar to that for the nonstratified analyses (results not shown). Therefore, we report the associations for both sexes combined. Significant negative associations of periodontitis were found with PF, RP, VT, SF, and MH. The adjusted regression coefficients of severe periodontitis in the multivariable models, compared with no/mild periodontitis, were −14.2 (95% confidence interval [CI] = −24.2 to −4.3) for PF, −13.0 (95% CI = −25.2 to −0.7) for RP, −6.3 (95% CI = −12.4 to −0.2) for VT, −7.5 (95% CI= −14.3 to −0.6) for SF, and −6.8 (95% CI = −12.6 to −1.0) for MH. These results suggest that severe periodontitis was significantly and independently associated with lower scores of SF‐36 for each of these five health status scales. In addition, in the multivariable model, advanced age was associated with decreases in PF and RP, longer duration of hemodialysis was associated with decreases in bodily pain and MH, diabetes was associated with a decrease in GH and MH, depression was associated with decreases in SF and MH, ischemic heart disease was associated with decreases in RP and RE, stroke was associated with decreases in SF and MH, hypoalbuminemia was associated with decreases in PF, VT, SF, RE, and MH, and higher BMI was associated with an increase in GH (Appendix Table 2).

**Table 2:** Crude and adjusted associations of periodontal health status with SF‐36 health scales (*n* = 188)

Periodontitis (vs. no/mild)	SF‐36 health scales							
	Crude	Adjusted^a^	Crude	Adjusted^a^	Crude	Adjusted^a^	Crude	Adjusted^a^
	PF		RP		BP		GH	
Moderate	**−**8.1 (−14.5, −1.7)*	−2.4 (−8.5, 3.6)	−7.0 (−14.1, 0.2)	−2.8 (−10.3, 4.7)	−1.7 (−5.2, 1.9)	−0.4 (−4.2, 3.4)	−3.9 (−7.0, −0.7)*	−3.1 (−6.5, 0.3)
Severe	−16.4 (−27.3, −5.6)*	−14.2 (−24.2, −4.3)*	−15.5 (−27.7, −3.4)*	−13.0 (−25.2, −0.7)*	−1.5 (−7.6, 4.5)	−1.9 (−8.1, 4.3)	−2.2 (−7.5, 3.2)	−1.2 (−6.8, 4.4)
	VT		SF		RE		MH	
Moderate	−4.1 (−7.5, −0.7)*	−3.1 (−6.8, 0.7)	−2.4 (−6.4, 1.6)	−1.3 (−5.5, 2.9)	−7.5 (−14.0, −1.0)*	−3.3 (−10.1, 3.5)	−0.9 (−4.2, 2.5)	0.4 (−3.1, 4.0)
Severe	−7.0 (−12.9, −1.2)*	−6.3 (−12.4, −0.2)*	−9.4 (−16.2, −2.7)*	−7.5 (−14.3, −0.6)*	−11.7 (−22.7, −0.6)*	−8.7 (−19.9, 2.4)	−8.2 (−13.9, −2.6)*	−6.8 (−12.6, −1.0)*

*Note*. Numbers in the table represent parameter estimates (95% confidence intervals) for each SF‐36 scale in the model. BP = bodily pain; GH = general health; MH = mental health; PF = physical functioning; RE = role emotional; RP = role physical; SF = social functioning; SF‐36 = Medical Outcome Study Short‐Form 36‐item health survey; VT = vitality.

a Adjusted for age, sex, underlying cause of hemodialysis, duration of hemodialysis, comorbidity, serum biomarkers, body mass index, smoking status, and alcohol consumption.

* *P* < 0.05.

## 4 DISCUSSION

All eight scales of the SF‐36 reported by the study participants were significantly lower than Japanese norms, which indicated hemodialysis has a negative impact on HRQoL. These findings are in line with previous studies (Evans et al., 1985; Merkus et al., 1997; Perlman et al., 2005). Diabetic nephropathy is the primary cause of end‐stage renal disease requiring dialysis (Akizawa, 2010). Hemodialysis patients are at risk of developing other medical conditions including malnutrition, depression, and cardiovascular disease (Akizawa, 2010; O'Connor & Corcoran, 2012). These health problems can adversely affect patients' activities of daily living and HRQoL. Disability in activities of daily living is highly prevalent in individuals with end‐stage renal disease compared with the general population (McAdams‐Demarco et al., 2012). The lower PF score of SF‐36 in this study population compared with the Japanese general population indicates that they perceived limitations in physical activities.

Periodontitis was found to be significantly associated with HRQoL among hemodialysis patients, even after adjusting for other important health characteristics. Study participants with severe periodontitis, compared with those with no/mild periodontitis, had worse scores in SF‐36 health scales of PF, RP, VT, SF, and MH, which suggests that hemodialysis patients with severe periodontitis perceived limitations in performing physical activities, problems with daily activities as a result of physical health, fatigue, interference with normal social activities due to physical and emotional problems, and psychological distress. To the best of our knowledge, this is the first study to demonstrate an association between clinical periodontal status and generic HRQoL measures in hemodialysis patients.

Decreased kidney function is associated with an immune dysfunction state that is known to contribute to the high prevalence of infections among these individuals (Kato et al., 2008). Thus, decreased kidney function may predispose to periodontitis, a chronic bacterial infection/overgrowth (Fisher, Taylor, West, & McCarthy, 2011). Moreover, kidney failure leads to metabolic bone disease. It is known that decreased kidney function increases osteoclast activity leading to bone turnover, and this may influence bone metabolic parameters (Couttenye et al., 1999). These pathophysiological changes may also explain the potential association between kidney failure and periodontitis.

Previous studies have demonstrated that poor oral health status had negative impacts on HRQoL (Naito et al., 2010; Ng & Leung, 2006; Sandberg & Wikblad, 2003; Zhou et al., 2011), and findings from the current study agree with these results. People with periodontitis are at increased risk of oral pain and tooth loss, which leads to limited oral function such as eating, swallowing, and speaking (Furuta & Yamashita, 2013). Poor oral health is related to decreased functional ability and physical fitness and decreased dietary intakes and variety (Hamalainen, Rantanen, Keskinen, & Meurman, 2004; Iwasaki et al., 2014; Iwasaki et al., 2015). Periodontitis also may cause halitosis, which can negatively impact on daily life activities such as socializing and communication with other people (Azodo, Onyeagba, & Odai, 2011). In addition, periodontitis and tooth loss causes embarrassment, negatively affecting appearance and self‐esteem (Petersen & Yamamoto, 2005; Abrahamsson, Wennstrom, & Hallberg, 2008), and can restrict social interactions. Furthermore, large epidemiological studies indicate that poor oral health could have an adverse effect on general health in people with reduced kidney function. For instance, periodontitis and tooth loss were demonstrated to be associated with higher mortality (Palmer et al., 2015; Ricardo et al., 2015). These problems related to periodontitis can have negative impacts on perceived general health and be reflected in deterioration in HRQoL measured by means of the SF‐36. Unfortunately, our study had a cross‐sectional design, which prevented us from assessing a temporal relationship and establishing causality.

There are several other limitations to the present study. First, because other information previously recognized as relevant to HRQoL, such as education, income, and social activity (Brennan, Williams, Berk, & Pasco, 2013; Nilsson, Rana, & Kabir, 2006), was not collected, a number of other potentially important confounders could not be included in the analyses. Residual confounding remains a risk. Second, our study participants were recruited at a single medical institution so that extrapolation of the findings to other populations is limited. Third, even though all non‐third molar teeth present were examined, periodontal health was assessed by partial mouth examination of only two buccal sites per tooth. Therefore, it is likely that the prevalence of periodontitis was underestimated, as some researchers have expressed concern over the discrepancy between a partial mouth examination and the “gold‐standard” full‐mouth examination (Eke, Thornton‐Evans, Wei, Borgnakke, & Dye, 2010). This potentially gross underestimation is due to the fact that periodontal tissue breakdown is site specific and does not occur symmetrically within the dentition nor even around a tooth, and hence prevents any reliable statistical weighting of the measurements recorded to accurately estimate the actual presence of periodontitis. On the other hand, despite such purported underestimation and misclassification of periodontitis as cases were categorized in less severe classes, our analyses still identified significant associations that probably would have been even more pronounced with measures from a gold‐standard periodontal examination. This would further support the concept of strong associations between periodontal health and HRQoL.

In summary, periodontitis, especially its severe form, is significantly associated with decreased HRQoL among hemodialysis patients. These findings support the importance of good oral health for general HRQoL in hemodialysis patients. Future multicenter, longitudinal studies that apply the gold‐standard periodontal examination protocol as well as collect more information on potential confounders are needed.

## Supporting information

Appendix Table 1. Interpretation of low and high scores of the 8 health scales of SF‐36^a^
Appendix Table 2.1. Associations of periodontal health status with SF‐36 health scales: PF and RP (covariates are presented)
Appendix Table 2.2. Associations of periodontal health status with SF‐36 health scales: BP and GH (covariates are presented)
Appendix Table 2.3. Associations of periodontal health status with SF‐36 health scales: VT and SF (covariates are presented)
Appendix Table 2.4. Associations of periodontal health status with SF‐36 health scales: RE and MH (covariates are presented)

Data S1. Supporting info item

## References

[cre250-bib-0032] Abrahamsson, K. H. , Wennstrom, J. L. , & Hallberg, U. (2008). Patients' views on periodontal disease; attitudes to oral health and expectancy of periodontal treatment: A qualitative interview study. Oral Health & Preventive Dentistry, 6(3), 209–216. 19119575

[cre250-bib-0020] Akizawa, T. (2010). Current status of dialysis therapy and related clinical guidelines in Japan. Japan Medical Association Journal, 53(3), 185–187.

[cre250-bib-0030] Azodo, C. C. , Onyeagba, M. I. , & Odai, C. D. (2011). Does concern about halitosis influence individual's oral hygiene practices? Nigerian Medical Journal, 52(4), 254–259. 22529509 10.4103/0300-1652.93799 PMC3329096

[cre250-bib-0035] Brennan, S. L. , Williams, L. J. , Berk, M. , & Pasco, J. A. (2013). Socioeconomic status and quality of life in population‐based Australian men: Data from the Geelong Osteoporosis Study. Australian and New Zealand Journal of Public Health, 37(3), 226–232. 23731104 10.1111/1753-6405.12063

[cre250-bib-0007] Buset, S. , Walter, C. , Friedmann, A. , Weiger, R. , Borgnakke, W. S. , & Zitzmann, N. U. (2016). Are periodontal diseases really silent? A systematic review of their effect on quality of life. Journal of Clinical Periodontology, 43(4), 333–344. 26810308 10.1111/jcpe.12517

[cre250-bib-0006] Chen, M. S. , & Hunter, P. (1996). Oral health and quality of life in New Zealand: A social perspective. Social Science & Medicine (1982), 43(8), 1213–1222. 8903125 10.1016/0277-9536(95)00407-6

[cre250-bib-0025] Couttenye, M. M. , D'Haese, P. C. , Verschoren, W. J. , Behets, G. J. , Schrooten, I. , & De Broe, M. E. (1999). Low bone turnover in patients with renal failure. Kidney International. Supplement, 73, S70–S76. 10633468 10.1046/j.1523-1755.1999.07308.x

[cre250-bib-0037] Eke, P. I. , Thornton‐Evans, G. O. , Wei, L. , Borgnakke, W. S. , & Dye, B. A. (2010). Accuracy of NHANES periodontal examination protocols. Journal of Dental Research, 89(11), 1208–1213. 20858782 10.1177/0022034510377793

[cre250-bib-0017] Evans, R. W. , Manninen, D. L. , Garrison, L. P. Jr. , Hart, L. G. , Blagg, C. R. , Gutman, R. A. , … Lowrie, E. G. (1985). The quality of life of patients with end‐stage renal disease. The New England Journal of Medicine, 312(9), 553–559. 3918267 10.1056/NEJM198502283120905

[cre250-bib-0024] Fisher, M. A. , Taylor, G. W. , West, B. T. , & McCarthy, E. T. (2011). Bidirectional relationship between chronic kidney and periodontal disease: A study using structural equation modeling. Kidney International, 79(3), 347–355. 20927035 10.1038/ki.2010.384 PMC3045269

[cre250-bib-0015] Fukuhara, S. , Ware, J. E. Jr. , Kosinski, M. , Wada, S. , & Gandek, B. (1998). Psychometric and clinical tests of validity of the Japanese SF‐36 health survey. Journal of Clinical Epidemiology, 51(11), 1045–1053. 9817122 10.1016/s0895-4356(98)00096-1

[cre250-bib-0016] Fukuhara, S ., Suzukamo, Y . (2004). Manual of SF‐36v2 Japanese version. Institute for Health Outcomes & Process Evaluation Research, Kyoto.

[cre250-bib-0026] Furuta, M. , & Yamashita, Y. (2013). Oral health and swallowing problems. Current Physical Medicine and Rehabilitation Reports, 1, 216–222. 24392281 10.1007/s40141-013-0026-x PMC3873078

[cre250-bib-0027] Hamalainen, P. , Rantanen, T. , Keskinen, M. , & Meurman, J. H. (2004). Oral health status and change in handgrip strength over a 5‐year period in 80‐year‐old people. Gerodontology, 21(3), 155–160. 15369018 10.1111/j.1741-2358.2004.00022.x

[cre250-bib-0005] Hollister, M. C. , & Weintraub, J. A. (1993). The association of oral status with systemic health, quality of life, and economic productivity. Journal of Dental Education, 57(12), 901–912. 8263237

[cre250-bib-0028] Iwasaki, M. , Taylor, G. W. , Manz, M. C. , Yoshihara, A. , Sato, M. , Muramatsu, K. , … Miyazaki, H. (2014). Oral health status: Relationship to nutrient and food intake among 80‐year‐old Japanese adults. Community Dentistry and Oral Epidemiology, 42(5), 441–450. 25353039 10.1111/cdoe.12100

[cre250-bib-0029] Iwasaki, M. , Kimura, Y. , Yoshihara, A. , Ogawa, H. , Yamaga, T. , Takiguchi, T. , … Matsubayashi, K. (2015). Association between dental status and food diversity among older Japanese. Community Dental Health, 32(2), 104–110. 26263604

[cre250-bib-0004] Kandelman, D. , Petersen, P. E. , & Ueda, H. (2008). Oral health, general health, and quality of life in older people. Special Care in Dentistry, 28(6), 224–236. 19068063 10.1111/j.1754-4505.2008.00045.x

[cre250-bib-0023] Kato, S. , Chmielewski, M. , Honda, H. , Pecoits‐Filho, R. , Matsuo, S. , Yuzawa, Y. , … Lindholm, B. (2008). Aspects of immune dysfunction in end‐stage renal disease. Clinical Journal of the American Society of Nephrology, 3(5), 1526–1533. 18701615 10.2215/CJN.00950208 PMC4571158

[cre250-bib-0013] Lowrie, E. G. , Curtin, R. B. , LePain, N. , & Schatell, D. (2003). Medical Outcomes Study Short Form‐36: A consistent and powerful predictor of morbidity and mortality in dialysis patients. American Journal of Kidney Diseases, 41(6), 1286–1292. 12776282 10.1016/s0272-6386(03)00361-5

[cre250-bib-0022] McAdams‐Demarco, M. A. , Law, A. , Garonzik‐Wang, J. M. , Gimenez, L. , Jaar, B. G. , Walston, J. D. , & Segev, D. L. (2012). Activity of daily living disability and dialysis mortality: Better prediction using metrics of aging. Journal of the American Geriatrics Society, 60(10), 1981–1982. 23057455 10.1111/j.1532-5415.2012.04161.x PMC4580268

[cre250-bib-0018] Merkus, M. P. , Jager, K. J. , Dekker, F. W. , Boeschoten, E. W. , Stevens, P. , & Krediet, R. T. (1997). Quality of life in patients on chronic dialysis: Self‐assessment 3 months after the start of treatment. The Necosad Study Group. American Journal of Kidney Diseases, 29(4), 584–592. 9100049 10.1016/s0272-6386(97)90342-5

[cre250-bib-0008] Naito, T. , Naito, M. , Miyaki, K. , Sugiyama, S. , Fujiki, S. , Habu, S. , … Nakayama, T. (2010). Oral health on the quality of life of dental patients: A cross‐sectional survey among dental patients attending private practices in Japan. Journal Fkuoka Dental College, 36(4), 139–147.

[cre250-bib-0036] Nilsson, J. , Rana, A. K. , & Kabir, Z. N. (2006). Social capital and quality of life in old age: Results from a cross‐sectional study in rural Bangladesh. Journal of Aging and Health, 18(3), 419–434. 16648394 10.1177/0898264306286198

[cre250-bib-0010] Ng, S. K. , & Leung, W. K. (2006). Oral health‐related quality of life and periodontal status. Community Dentistry and Oral Epidemiology, 34(2), 114–122. 16515675 10.1111/j.1600-0528.2006.00267.x

[cre250-bib-0021] O'Connor, N. R. , & Corcoran, A. M. (2012). End‐stage renal disease: Symptom management and advance care planning. American Family Physician, 85(7), 705–710. 22534347

[cre250-bib-0014] Page, R. C. , & Eke, P. I. (2007). Case definitions for use in population‐based surveillance of periodontitis. Journal of Periodontology, 78(7 Suppl), 1387–1399. 17608611 10.1902/jop.2007.060264

[cre250-bib-0033] Palmer, S. C. , Ruospo, M. , Wong, G. , Craig, J. C. , Petruzzi, M. , De Benedittis, M. , … ORAL‐D Study Investigators . (2015). Dental health and mortality in people with end‐stage kidney disease treated with hemodialysis: A multinational cohort study. American Journal of Kidney Diseases, 66(4), 666–676. 26120038 10.1053/j.ajkd.2015.04.051

[cre250-bib-0019] Perlman, R. L. , Finkelstein, F. O. , Liu, L. , Roys, E. , Kiser, M. , Eisele, G. , … Saran, R. (2005). Quality of life in chronic kidney disease (CKD): A cross‐sectional analysis in the Renal Research Institute‐CKD study. American Journal of Kidney Diseases, 45(4), 658–666. 15806468 10.1053/j.ajkd.2004.12.021

[cre250-bib-0031] Petersen, P. E. , & Yamamoto, T. (2005). Improving the oral health of older people: The approach of the WHO Global Oral Health Programme. Community Dentistry and Oral Epidemiology, 33(2), 81–92. 15725170 10.1111/j.1600-0528.2004.00219.x

[cre250-bib-0034] Ricardo, A. C. , Athavale, A. , Chen, J. , Hampole, H. , Garside, D. , Marucha, P. , & Lash, J. P. (2015). Periodontal disease, chronic kidney disease and mortality: Results from the third National Health and Nutrition Examination Survey. BMC Nephrology, 16, 97. 26149680 10.1186/s12882-015-0101-x PMC4492086

[cre250-bib-0011] Sandberg, G. E. , & Wikblad, K. F. (2003). Oral health and health‐related quality of life in type 2 diabetic patients and non‐diabetic controls. Acta Odontologica Scandinavica, 61(3), 141–148. 12868687 10.1080/00016350310002559

[cre250-bib-0002] Sharma, P. , Dietrich, T. , Sidhu, A. , Vithlani, V. , Rahman, M. , Stringer, S. , … Chapple, I. L. (2014). The periodontal health component of the Renal Impairment In Secondary Care (RIISC) cohort study: A description of the rationale, methodology and initial baseline results. Journal of Clinical Periodontology, 41(7), 653–661. 24738870 10.1111/jcpe.12263

[cre250-bib-0003] Teratani, G. , Awano, S. , Soh, I. , Yoshida, A. , Kinoshita, N. , Hamasaki, T. , … Ansai, T. (2013). Oral health in patients on haemodialysis for diabetic nephropathy and chronic glomerulonephritis. Clinical Oral Investigations, 17(2), 483–489. 22552594 10.1007/s00784-012-0741-1

[cre250-bib-0001] Wakasugi, M. , Kazama, J. J. , & Narita, I. (2015). Anticipated increase in the number of patients who require dialysis treatment among the aging population of Japan. Therapeutic Apheresis and Dialysis, 19(3), 201–206. 25545737 10.1111/1744-9987.12266

[cre250-bib-0009] Wang, T. F. , Chou, C. , & Yu, S. (2013). Assessing the effects of oral health‐related variables on quality of life in Taiwanese adults. Quality of Life Research, 22(4), 811–825. 22644543 10.1007/s11136-012-0205-8

[cre250-bib-0012] Zhou, X. , Wang, Z. , Song, Y. , Zhang, J. , & Wang, C. (2011). Periodontal health and quality of life in patients with chronic obstructive pulmonary disease. Respiratory Medicine, 105(1), 67–73. 20630736 10.1016/j.rmed.2010.06.017

