# Supplementary material for: Periodontitis and health‐related quality of life in hemodialysis patients
Source: Clin Exp Dent Res. 2016 Nov 24;3(1):13–8. doi: 10.1002/cre2.50 (PMC5839240; doi:10.1002/cre2.50)
Supplement: Supplementary file 1 — Data S1. Supporting info item [file CRE2-3-13-s001.docx]

| Appendix Table 1. Interpretation of low and high scores of the 8 health scales of SF-36^a^ | | | |
| --- | --- | --- | --- |
|  | Scores | | |
|  | Very low |  | Very high |
| Physical functioning (PF) | Very limited in performing all physical activities, including bathing or dressing. |  | Performs all types of physical activities including the most vigorous without limitations due to health. |
| Role physical (RP) | Problems with work or other daily activities as a result of physical health. |  | No problems with work or other daily activities. |
| Bodily pain (BP) | Very severe and extremely limiting pain. |  | No pain or limitations due to pain. |
| General health (GH) | Evaluates personal health as poor and believes it is likely to get worse. |  | Evaluates personal health as excellent. |
| Vitality (VT) | Feels tired and worn out all of the time. |  | Feels full of pep and energy all of the time. |
| Social functioning (SF) | Extreme and frequent interference with normal social activities due to physical and emotional problems. |  | Performs normal social activities without interference due to physical or emotional problems. |
| Role emotional (RE) | Problems with work or other daily activities as a result of emotional problems. |  | No problems with work or other daily activities. |
| Mental health (MH) | Feelings of nervousness and depression all of the time. |  | Feels peaceful, happy, and calm all of the time. |
| ^a^Adapted from Fukuhara S, Suzukamo Y. *Manual of SF-36v2 Japanese version*. Institute for Health Outcomes & Process Evaluation Research, Kyoto, 2004 (16).  SF-36 = Medical Outcome Study Short-Form 36-item health survey. | | | |

| Appendix Table 2.1. Associations of periodontal health status with SF-36 health scales: PF and RP (covariates are presented) | | | | |
| --- | --- | --- | --- | --- |
|  | SF-36 health domains | | | |
|  | Physical functioning (PF) | | Role physical (RP) | |
|  | Crude model  (periodontitis only) | Multivariable model (fully adjusted) | Crude model  (periodontitis only) | Multivariable model (fully adjusted) |
| Periodontitis (vs. No/Mild) |  |  |  |  |
| Moderate | **-8.1 (-14.5, -1.7)** | -2.4 (-8.5, 3.6) | -7.0 (-14.1, 0.2) | -2.8 (-10.3, 4.7) |
| Severe | **-16.4 (-27.3, -5.6)** | **-14.2 (-24.2, -4.3)** | **-15.5 (-27.7, -3.4)** | **-13.0 (-25.2, -0.7)** |
| Age |  | **-0.5 (-0.8, -0.3)** |  | **-0.4 (-0.7, -0.1)** |
| Sex |  |  |  |  |
| Male (vs. female) |  | 4.7 (-2.5, 11.8) |  | 2.3 (-6.5, 11.1) |
| Underlying cause of hemodialysis (vs. other) |  |  |  |  |
| Diabetic nephropathy |  | -10.6 (-21.6, 0.4) |  | -9.3 (-23.0, 4.4) |
| Chronic glomerulonephritis |  | -1.2 (-8.5, 6.0) |  | 2.0 (-7.0, 11.0) |
| Duration of hemodialysis |  | -0.2 (-0.4, 0.1) |  | -0.1 (-0.3, 0.3) |
| Medical diagnosis (vs. negative) |  |  |  |  |
| Hypertension |  | 2.2 (-3.5, 8.0) |  | -1.8 (-8.9, 5.2) |
| Diabetes |  | 4.7 (-5.0, 14.5) |  | 2.7 (-9.4, 14.8) |
| Depression |  | -13.2 (-29.9, 3.5) |  | -13.6 (-34.4, 7.2) |
| Medical history (vs. negative) |  |  |  |  |
| Ischemic heart disease |  | -3.7 (-11.3, 3.9) |  | **-12.9 (-22.4, -3.4)** |
| Stroke |  | -5.5 (-12.1, 1.1) |  | -2.9 (-11.1, 5.4) |
| Abnormality in serum biomarkers (vs. Negative) |  |  |  |  |
| Albumin < 3.6 g/dL |  | **-11.4 (-17.3, -5.5)** |  | -5.8 (-13.0, 1.4) |
| Non-high-density lipoprotein cholesterol ≥ 150 mg/dL |  | 3.9 (-3.3, 11.2) |  | 2.7 (-6.5, 11.9) |
| BMI |  | 0.6 (-0.4, 1.7) |  | 0.4 (-0.9, 1.7) |
| Smoking status (vs. never smoked) |  |  |  |  |
| Previous smoker |  | 0.3 (-6.6, 7.3) |  | 4.8 (-3.8, 13.4) |
| Current smoker |  | -5.4 (-14.6, 3.9) |  | -4.1 (-15.5, 7.4) |
| Alcohol consumption |  |  |  |  |
| Ethanol ≥20 g/day (vs. negative) |  | -5.9 (-13.7, 1.9) |  | 3.8 (-6.0, 13.6) |
| Numbers in the table represent parameter estimates (95% confidence intervals) for each SF-36 scale in the model. | | | | |
| **Bold** text indicates statistically significant findings (*P*<0.05). | | | | |

| Appendix Table 2.2. Associations of periodontal health status with SF-36 health scales: BP and GH (covariates are presented) | | | | |
| --- | --- | --- | --- | --- |
|  | SF-36 health domains | | | |
|  | Bodily pain (BP) | | General health (GH) | |
|  | Crude model  (periodontitis only) | Multivariable model (fully adjusted) | Crude model  (periodontitis only) | Multivariable model (fully adjusted) |
| Periodontitis (vs. No/Mild) |  |  |  |  |
| Moderate | -1.7 (-5.2, 1.9) | -0.4 (-4.2, 3.4) | **-3.9 (-7.0, -0.7)** | -3.1 (-6.5, 0.3) |
| Severe | -1.5 (-7.6, 4.5) | -1.9 (-8.1, 4.3) | -2.2 (-7.5, 3.2) | -1.2 (-6.8, 4.4) |
| Age |  | -0.1 (-0.3, 0.1) |  | 0.1 (-0.1, 0.2) |
| Sex |  |  |  |  |
| Male (vs. female) |  | 4.3 (-0.2, 8.8) |  | -0.3 (-4.3, 3.8) |
| Underlying cause of hemodialysis (vs. others) |  |  |  |  |
| Diabetic nephropathy |  | -4.3 (-11.2, 2.6) |  | 4.5 (-1.7, 10.7) |
| Chronic glomerulonephritis |  | -0.9 (-5.5, 3.6) |  | -0.7 (-4.8, 3.4) |
| Duration of hemodialysis |  | **-0.2 (-0.4, -0.1)** |  | -0.1 (-0.2, 0.1) |
| Medical diagnosis (vs. negative) |  |  |  |  |
| Hypertension |  | -2.7 (-6.3, 0.9) |  | 1.9 (-1.3, 5.1) |
| Diabetes |  | 2.5 (-3.7, 8.6) |  | **-5.6 (-11.1, -0.1)** |
| Depression |  | -4.9 (-15.4, 5.6) |  | -0.1 (-9.5, 9.3) |
| Medical history (vs. negative) |  |  |  |  |
| Ischemic heart disease |  | -0.1 (-4.8, 4.8) |  | -3.5 (-7.8, 0.7) |
| Stroke |  | -1.7 (-5.9, 2.4) |  | 1.1 (-2.6, 4.8) |
| Abnormality in serum biomarkers (vs. Negative) |  |  |  |  |
| Albumin < 3.6 g/dL |  | -2.2 (-5.9, 1.5) |  | -2.6 (-5.9, 0.7) |
| Non-high-density lipoprotein cholesterol ≥ 150 mg/dL |  | 0.3 (-4.3, 4.8) |  | -1.6 (-5.7, 2.4) |
| BMI |  | -0.3 (-1.0, 0.3) |  | **0.7 (0.1, 1.2)** |
| Smoking status (vs. never smoked) |  |  |  |  |
| Previous smoker |  | -3.6 (-8.0, 0.7) |  | 1.5 (-2.4, 5.4) |
| Current smoker |  | -5.1 (-10.9, 0.7) |  | -1.5 (-6.7, 3.7) |
| Alcohol consumption |  |  |  |  |
| Ethanol ≥20 g/day (vs. negative) |  | -2.0 (-6.9, 2.9) |  | -2.1 (-6.5, 2.3) |
| Numbers in the table represent parameter estimates (95% confidence intervals) for each SF-36 scale in the model. | | | | |
| **Bold** text indicates statistically significant findings (*P*<0.05). | | | | |

| Appendix Table 2.3. Associations of periodontal health status with SF-36 health scales: VT and SF (covariates are presented) | | | | |
| --- | --- | --- | --- | --- |
|  | SF-36 health domains | | | |
|  | Vitality (VT) | | Social functioning (SF) | |
|  | Crude model  (periodontitis only) | Multivariable model (fully adjusted) | Crude model  (periodontitis only) | Multivariable model (fully adjusted) |
| Periodontitis (vs. No/Mild) |  |  |  |  |
| Moderate | **-4.1 (-7.5, -0.7)** | -3.1 (-6.8, 0.7) | -2.4 (-6.4, 1.6) | -1.3 (-5.5, 2.9) |
| Severe | **-7.0 (-12.9, -1.2)** | **-6.3 (-12.4, -0.2)** | **-9.4 (-16.2, -2.7)** | **-7.5 (-14.3, -0.6)** |
| Age |  | 0.1 (-0.1, 0.2) |  | 0.1 (-0.1, 0.2) |
| Sex |  |  |  |  |
| Male (vs. female) |  | -1.4 (-5.8, 3.0) |  | -2.8 (-7.8, 2.1) |
| Underlying cause of hemodialysis (vs. others) |  |  |  |  |
| Diabetic nephropathy |  | 3.2 (-3.2, 9.7) |  | -2.5 (-10.1, 5.1) |
| Chronic glomerulonephritis |  | 0.7 (-3.8, 5.2) |  | 0.4 (-4.6, 5.5) |
| Duration of hemodialysis |  | -0.1 (-0.2, 0.1) |  | 0.1 (-0.1, 0.2) |
| Medical diagnosis (vs. negative) |  |  |  |  |
| Hypertension |  | 1.3 (-2.3, 4.8) |  | -1.1 (-5.1, 2.9) |
| Diabetes |  | -2.5 (-8.2, 3.3) |  | -0.1 (-6.8, 6.7) |
| Depression |  | 3.2 (-7.1, 13.5) |  | **-21.8 (-33.3, -10.3)** |
| Medical history (vs. negative) |  |  |  |  |
| Ischemic heart disease |  | -4.3 (-9.0, 0.4) |  | -2.3 (-7.6, 2.9) |
| Stroke |  | -2.6 (-6.7, 1.4) |  | **-6.0 (-10.5, -1.4)** |
| Abnormality in serum biomarkers (vs. Negative) |  |  |  |  |
| Albumin < 3.6 g/dL |  | **-4.0 (-7.6, -0.4)** |  | **-4.5 (-8.5, -0.4)** |
| Non-high-density lipoprotein cholesterol ≥ 150 mg/dL |  | 1.7 (-2.8, 6.1) |  | -0.4 (-5.4, 4.6) |
| BMI |  | 0.2 (-0.5, 0.8) |  | 0.3 (-0.4, 1.1) |
| Smoking status (vs. never smoked) |  |  |  |  |
| Previous smoker |  | 3.2 (-1.0, 7.5) |  | 2.4 (-2.4, 7.2) |
| Current smoker |  | 0.7 (-5.0, 6.4) |  | 1.5 (-4.9, 7.8) |
| Alcohol consumption |  |  |  |  |
| Ethanol ≥20 g/day (vs. negative) |  | -1.1 (-6.0, 3.7) |  | 0.8 (-4.6, 6.2) |
| Numbers in the table represent parameter estimates (95% confidence intervals) for each SF-36 scale in the model. | | | | |
| **Bold** text indicates statistically significant findings (*P*<0.05). | | | | |

| Appendix Table 2.4. Associations of periodontal health status with SF-36 health scales: RE and MH (covariates are presented) | | | | |
| --- | --- | --- | --- | --- |
|  | SF-36 health domains | | | |
|  | Role emotional (RE) | | Mental health (MH) | |
|  | Crude model  (periodontitis only) | Multivariable model (fully adjusted) | Crude model  (periodontitis only) | Multivariable model (fully adjusted) |
| Periodontitis (vs. No/Mild) |  |  |  |  |
| Moderate | **-7.5 (-14.0, -1.0)** | -3.3 (-10.1, 3.5) | -0.9 (-4.2, 2.5) | 0.4 (-3.1, 4.0) |
| Severe | **-11.7 (-22.7, -0.6)** | -8.7 (-19.9, 2.4) | **-8.2 (-13.9, -2.6)** | **-6.8 (-12.6, -1.0)** |
| Age |  | -0.1 (-0.4, 0.1) |  | 0.1 (-0.1, 0.2) |
| Sex |  |  |  |  |
| Male (vs. female) |  | 4.0 (-4.1, 12.0) |  | 0.2 (-4.0, 4.4) |
| Underlying cause of hemodialysis (vs. others) |  |  |  |  |
| Diabetic nephropathy |  | 1.6 (-10.8, 14.0) |  | 4.9 (-1.2, 11.0) |
| Chronic glomerulonephritis |  | 2.8 (-5.4, 11.0) |  | 1.0 (-3.2, 5.3) |
| Duration of hemodialysis |  | 0.1 (-0.2, 0.4) |  | **-0.2 (-0.3, -0.1)** |
| Medical diagnosis (vs. negative) |  |  |  |  |
| Hypertension |  | -3.1 (-9.6, 3.4) |  | -2.0 (-5.4, 1.3) |
| Diabetes |  | -4.7 (-15.8, 6.3) |  | **-6.8 (-12.2, -1.3)** |
| Depression |  | -17.7 (-36.4, 1.1) |  | **-13.3 (-23.0, -3.6)** |
| Medical history (vs. negative) |  |  |  |  |
| Ischemic heart disease |  | **-10.0 (-18.6, -1.4)** |  | 0.5 (-3.9, 5.0) |
| Stroke |  | -6.4 (-13.9, 1.0) |  | **-5.6 (-9.4, -1.7)** |
| Abnormality in serum biomarkers (vs. Negative) |  |  |  |  |
| Albumin < 3.6 g/dL |  | **-8.2 (-14.8, -1.6)** |  | **-3.5 (-6.9, -0.1)** |
| Non-high-density lipoprotein cholesterol ≥ 150 mg/dL |  | 1.7 (-6.4, 9.9) |  | -2.8 (-7.0, 1.4) |
| BMI |  | -0.7 (-1.9, 0.5) |  | 0.1 (-0.6, 0.6) |
| Smoking status (vs. never smoked) |  |  |  |  |
| Previous smoker |  | 0.1 (-7.7, 7.9) |  | 1.2 (-2.9, 5.2) |
| Current smoker |  | -5.6 (-16.0, 4.8) |  | -2.4 (-7.8, 3.0) |
| Alcohol consumption |  |  |  |  |
| Ethanol ≥20 g/day (vs. negative) |  | 2.7 (-6.1, 11.5) |  | 0.9 (-3.7, 5.4) |
| Numbers in the table represent parameter estimates (95% confidence intervals) for each SF-36 scale in the model. | | | | |
| **Bold** text indicates statistically significant findings (*P*<0.05). | | | | |
